# Non-Rapid Eye Movement Sleep Parasomnias and Migraine: A Role of Orexinergic Projections

**DOI:** 10.3389/fneur.2018.00095

**Published:** 2018-02-28

**Authors:** Antonietta Messina, Ilaria Bitetti, Francesco Precenzano, Diego Iacono, Giovanni Messina, Michele Roccella, Lucia Parisi, Margherita Salerno, Anna Valenzano, Agata Maltese, Monica Salerno, Francesco Sessa, Giuseppe Davide Albano, Rosa Marotta, Ines Villano, Gabriella Marsala, Christian Zammit, Francesco Lavano, Marcellino Monda, Giuseppe Cibelli, Serena Marianna Lavano, Beatrice Gallai, Roberto Toraldo, Vincenzo Monda, Marco Carotenuto

**Affiliations:** ^1^Department of Experimental Medicine, University of Campania “Luigi Vanvitelli”, Naples, Italy; ^2^Clinic of Child and Adolescent Neuropsychiatry, Center for Childhood Headache, Department of Mental Health, Physical and Preventive Medicine, University of Campania “Luigi Vanvitelli”, Naples, Italy; ^3^Neurodevelopmental Research Lab, Biomedical Research Institute of New Jersey (BRInj), Cedar Knolls NJ, United States; ^4^Neuroscience Research, MidAtlantic Neonatology Associates, Atlantic Health System, Morristown NJ, United States; ^5^Neuropathology Research, MidAtlantic Neonatology Associates (MANA) and Biomedical Research Institute of New Jersey (BRInj), Morristown, NJ, United States; ^6^Department of Clinical and Experimental Medicine, University of Foggia, Foggia, Italy; ^7^Child Neuropsychiatry, Department of Psychology and Pedagogical Sciences, University of Palermo, Palermo, Italy; ^8^Department of Health Sciences, University “Magna Graecia”, Catanzaro, Italy; ^9^Struttura Complessa di Farmacia, Azienda Ospedaliero-Universitaria, Ospedali Riuniti di Foggia, Foggia, Italy; ^10^Anatomy Department, Faculty of Medicine and Surgery, University of Malta, Msida, Malta; ^11^Department of Surgical and Biomedical Sciences, University of Perugia, Perugia, Italy

**Keywords:** serotonergic system, orexinergic system, sleep–wake rhythm, migraine, pro-inflammatory peptides

## Abstract

**Introduction:**

Sleep and migraine share a common pathophysiological substrate, although the underlying mechanisms are unknown. The serotonergic and orexinergic systems are both involved in the regulation of sleep/wake cycle, and numerous studies show that both are involved in the migraine etiopathogenesis. These two systems are anatomically and functionally interconnected. Our hypothesis is that in migraine a dysfunction of orexinergic projections on the median raphe (MR) nuclei, interfering with serotonergic regulation, may cause Non-Rapid Eye Movement parasomnias, such as somnambulism.

**Hypothesis/theory:**

Acting on the serotonergic neurons of the raphe nuclei, the dysfunction of orexinergic neurons would lead to a higher release of serotonin. The activation of serotonergic receptors located on the walls of large cerebral vessels would lead to abnormal vasodilatation and consequently increase transmural pressure. This process could activate the trigeminal nerve terminals that innervate vascular walls. As a consequence, there is activation of sensory nerve endings at the level of hard vessels in the meninges, with release of pro-inflammatory peptides (e.g., substance P and CGRP). Within this hypothetical frame, the released serotonin could also interact with trigeminovascular afferents to activate and/or facilitate the release of the neuropeptide at the level of the trigeminal ganglion. The dysregulation of the physiological negative feedback of serotonin on the orexinergic neurons, in turn, would contribute to an alteration of the whole system, altering the sleep–wake cycle.

**Conclusion:**

Serotonergic neurons of the MR nuclei receive an excitatory input from hypothalamic orexin/hypocretin neurons and reciprocally inhibit orexin/hypocretin neurons through the serotonin 1A receptor (or 5-HT1A receptor). Considering this complex system, if there is an alteration it may facilitate the pathophysiological mechanisms involved in the migraine, while it may produce at the same time an alteration of the sleep–wake rhythm, causing sleep disorders such as sleepwalking. Understanding the complex mechanisms underlying migraine and sleep disorders and how these mechanisms can interact with each other, it would be crucial to pave the way for new therapeutic strategies.

## Introduction/Background of Migraine and Non-Rapid Eye Movement (NREM) Parasomnias

Sleep and migraine share a common pathophysiological substrate. While the mechanisms by which sleep deprivation or sleep dysfunction leads to headache are unknown, their association has been recognized for decades. The frontal aching headaches can develop both in normal subjects and in those who suffer from tension-type headaches who are deprived of sleep (1, 2).

The serotonergic system, well known as being central in the migraine attacks, demonstrates circadian and circannual rhythmicity. It is under control of the central nervous system (CNS), like other biorhythms (e.g., blood pressure). During the onset of a migraine attack, the urinary excretion of 5-hydroxyindoleacetic acid (5-HIAA)—the main metabolite of serotonin—increases while platelet 5-hydroxytryptamine (5-HT) rapidly decreases. 5-HT pharmacologic depletion can induce a migraine attack and intravenous 5-HT can stop acute migraine attacks as well. Alike, during REM sleep when the dorsal raphe (DR) nucleus is silent, the systemic serotonin decreases. In part, this could explain the relationship between REM sleep and migraine. In addition, their relationship is also explained by the relationship between the hypothalamus (with other crucial regions involved in sleep regulation) and the areas engaged in nociception and migraine pathogenesis. These findings suggest that dysfunction in the sleep-regulating systems could generate headaches. Sleep loss may worsen pain, but not sleep fragmentation. As previously described in healthy mice, sleep loss increases the sensitivity to noxious stimuli without sensory hyperresponsiveness (3, 4). Moreover, the role of melatonin MT2 receptors in the antinociception modulation through the glutamatergic pathway (5) could also be considered and that melatonin levels are lower in the migraine sufferers regardless of the age, so suggesting its potential role as preventive therapy both for nociception modulation and sleep cycle regulation (6–12).

Headache and sleep have some anatomical and functional in common. The incidence of somnambulism (sleepwalking) may be increased in children with migraine (13, 14), and it has been proposed as a minor diagnostic criterion for migraine. On the other hand, serotonergic neurotransmission is involved during slow wave sleep (SWS), playing a predominant role in the migraine mechanisms. Clinical similarities such as genetic predisposition or reactivity to external stimuli suggest that migraine and somnambulism could have similar predisposing factors and perhaps common physiologic pathways. Medications used to treat migraine, such as propranolol (15) or amitriptyline (16), have sometimes induced, but most often cured, sleepwalking or other NREM parasomnias (17–19). In this hypothesis paper, we do not suggest that migraine headache and somnambulism may be part of the same pathology, but that migraine and somnambulism may follow a common pathway, either chemical or topographic (20, 21).

Several reports have described the association between migraine and NREM parasomnias in the pediatric age. Barabas et al. (13) performed the first study describing a correlation between these two disorders. Analyzing 4 groups of patients (60 with migraine, 42 with non-migraine headache, 60 with epilepsy, and 60 with learning disabilities/neurologic impairment), they found at least 2 episodes of somnambulism in 30% of migraineurs vs. 4.8% of those with non-migraine headaches, 5% of those with learning disabilities/neurologic impairment, and 6.6% of epileptics. Other studies confirmed these data. Pradalier et al. (15) found an incidence of sleepwalking in 21.9% of migraine subjects vs. 6.6% of controls. Giroud et al. (14) found a history of somnambulism in 29.4% of migraine subjects vs. 5.4% of non-migraine headache subjects. The analysis of different types of migraine showed that the highest prevalence was found in the ophthalmic migraine (70%), in common migraine (24%), and in classic migraine (20%). Noticing that the somnambulism appeared before migraine, those authors hypothesized that this sleep disorder and migraine could be a different age-related expression of the same neurotransmitter imbalance, probably of the serotonergic axis. The actual classification of headache disorders ICHD-3 (22) includes sleepwalking, sleep talking, night terrors, and bruxism among the additional conditions that may also occur in patients affected by migraine independently on sex and age (code 1.6). This suggests a close relationship between sleep disorders and migraine, particularly with disorders of arousal/NREM parasomnias (2, 23–30). In a study published in 1986 (31), Dexter asked to the parents of 100 migrainous patients about the occurrence of these disturbances (such as night terrors, sleepwalking, and nocturnal enuresis in the first 2 decades of life). Dexter found an incidence of 71% for night terrors/pavor nocturnus (vs. 11% of controls), 55% of somnambulism (vs. 16% of controls), and 41% of nocturnal enuresis (vs. 16% of controls). These findings were similar to those described by Giroud in 1986 (14) and by Miller in 2003 (32), while another study failed to confirm these findings data (33).

Sleepwalking has been associated with migraine. Somnambulism and migraine can appear at different ages, the former during late infancy, the latter during childhood, and both could be linked to a different age-related expression of a serotonergic metabolic dysfunction (27), which could occur independently on sleep-disordered breathing. Instead, when both conditions are present, the association may be linked to the hypercapnic acidosis, which leads to a stimulation of serotonergic neurons, resulting in an increased excitability of motoneurons directing to the somnambulism (27, 34).

The relationship between sleep disturbance and migraine/headache could involve another fundamental neurobiological system, the orexin/hypocretin system, which seems to play a key role in regulating both the sleep/wake cycle and REM sleep (35), and it may also be associated with migraine pathogenesis. Indeed, orexin/hypocretin A and orexin/hypocretin B are hypothalamic excitatory neuropeptides that, in addition to regulating sleep/wake rhythm, play a role in many biological pathways involved in thermoregulation, energy metabolism control, mood and emotional regulation, energy homeostasis, reward mechanisms, drug dependence, cardiovascular responses, sexual behavior, nutritional behavior, and spontaneous physical activity (36–38). It is interesting to note that some symptoms associated with migraine such as tiredness, yawning, drowsiness, and desire for certain foods may be due to an involvement of the orexin/hypocretin system. In mammals, orexin/hypocretin A and orexin/hypocretin B are both synthesized from pre–pro-orexin in hypothalamic and central areas (39, 40). The orexin/hypocretin peptides influence two specific receptors: orexin/hypocretin 1-receptor (OX1R), localized in prefrontal cortex and infralimbic, hippocampus, amygdala, stria terminalis bed nucleus, paraventricular thalamus, front hypothalamus, median raphe (MR) nucleus, ventral tegmental area/pedunculopontine nucleus (41, 42), and orexin/hypocretin 2-receptor (OX2R), localized at amygdala, tuberomammillary nucleus, Arc, dorsomedian hypothalamic nucleus, locus coeruleus and laterodorsal tegmental nucleus, lateral hypothalamus, stria terminalis bed nucleus, paraventricular thalamus, DR, ventral tegmental area/pedunculopontine nucleus, hippocampus, and median septal nucleus (42). The activity of orexin/hypocretin neurons is modulated by several neurotransmitters: GABA (43), noradrenaline, and serotonin inhibit the activity of orexin/hypocretin neurons (44); glutamate (45), cholecystokinin, neurotensin, oxytocin, and vasopressin seem to have instead an excitatory action on orexin neurons (46–48). The complex mechanisms by which the orexin/hypocretin system interacts with other brain systems and with the whole organism and the roles that these interactions may play are still to be clarified.

## Hypothesis/Theory

Neural pathways controlling sleep and pain are anatomically, physiologically, and neurochemically crossed. These neural systems are found in the brain, hypothalamus, and basal brain. The activity of the serotonergic nuclei of the cerebral trunk (MR nuclei) is physiologically reduced during REM sleep, and these structures are involved in anti-nociceptive control. About the main serotonergic system, the MR and DR nuclei provide parallel and overlapping projections to many forebrain structures with axon fibers exhibiting distinct structural and functional characteristics. Serotonin neurons within the rostral DR are uniquely interconnected with brain areas associated with emotion and motivation such as the amygdala, accumbens, and ventral pallidum nuclei. In contrast, the serotonin neurons in the MR are characterized by their dominion over the septum and hippocampus (49). Serotonin pathways have been found to be important in the migraine pathophysiology. One of the main sleep–wake rhythm control systems is the orexin/hypocretin system (50). Orexin/hypocretin neurons are localized in the lateral hypothalamus and give projections throughout the brain and spinal cord, densely innervating the DR nucleus and MR nucleus, which contain serotonergic neurons (51).

Our hypothesis is that in migraines a dysfunction of orexinergic projections on the MR nuclei, interfering with serotonergic regulation, may cause NREM parasomnias, such as somnambulism.

## Evaluation of the Hypothesis

Serotonin and orexin/hypocretin systems are anatomically and functionally interconnected. Serotonergic neurons receive excitatory input from hypothalamic orexin/hypocretin neurons and reciprocally inhibit orexin/hypocretin neurons through the 5HT1A receptor. It is possible that if this complex system is altered, it may facilitate the pathophysiological mechanisms involved in the migraine and on the other hand produce an alteration of the sleep–wake rhythm causing sleep disorders, such as sleepwalking. About the orexinergic innervation role in the migraine pathophysiology, axons immunoreactive to orexin/hypocretin A are reported as present at low density in lateral posterior (LP), and lateral dorsal (LD) nuclei of thalamus, and most median part of thalamic posterior complex (Po), and at very low density in ventral posterior median nucleus of thalamus (VPM) and most lateral part of Po (52). Moreover, if examined in sections containing the trigeminovascular neuron(s), low density of orexinergic immunopositive axons and varicosities appears to be close apposition to the proximal and distal dendrites but not the cell body (52, 53). These data show that the orexinergic axons originate mainly in the perifornical hypothalamic area. The orexin/hypocretin system originates in the LH and projects to the cortex, thalamus, brainstem, spinal cord, and other hypothalamic nuclei (39, 54–56). The wide distribution of orexin fibers in the brain supports a role in regulating food intake, arousal, wakefulness, and sympathetically mediated increase in body temperature, heart rate, sexual behavior, and blood pressure (57).

About the potential relevance for the pathophysiology of migraine, the orexinergic axons are in nociceptive laminae of the medullary dorsal horn and in close apposition to thalamic trigeminovascular neurons, although no data are yet available regarding the direction in which orexin/hypocretin may modulate the thalamic trigeminovascular neurons. In the migraine context, it is reasonable to hypothesize that the mechanism by which eating may reduce headache intensity involves not only local release of GABA from activated melanin-concentrating hormone (MCH)-expressing neurons but also inhibition of facilitatory orexin input to thalamic trigeminovascular neurons. These activities are induced by glucose level increase, considering that orexin/hypocretin neurons are inhibited by glucose (51, 58). The chemical pathway to modulate the activity of thalamic trigeminovascular neurons has been reported to be controlled mainly by glutamate, GABA, dopamine, and serotonin and in a minor level by noradrenaline and histamine, MCH, and orexin/hypocretin (52, 59).

**Figure 1:**
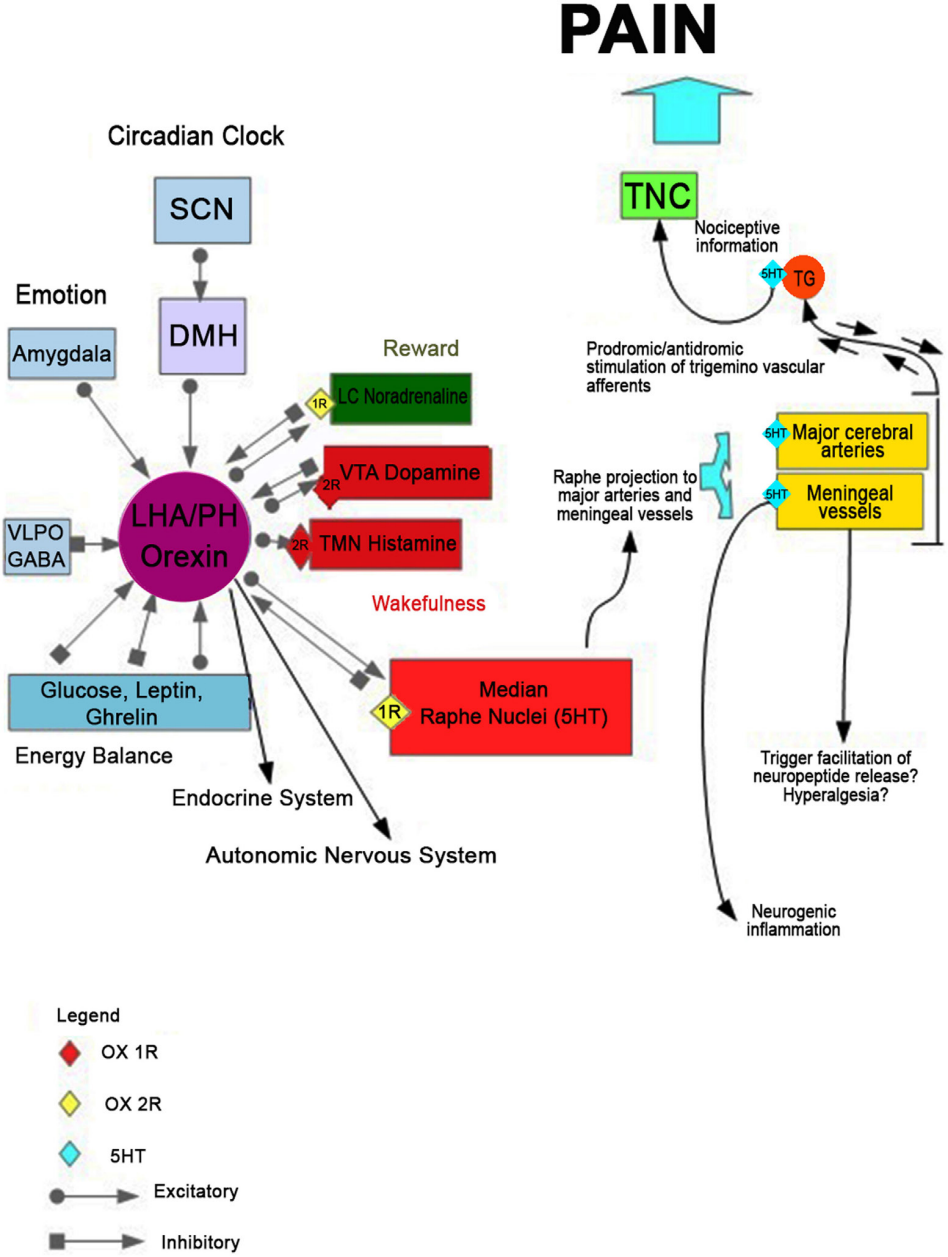
The hypothetical mechanism leading to the onset of migraine pain and disorder of the sleep/wake cycle. Disruption of orexinergic neurons would lead, acting on the serotonergic neurons of the raphe nuclei, to a massive release of serotonin from the median raphe nucleus. It could activate the serotonergic receptors located on the walls of large cerebral vessels, determining an abnormal vasodilatation and increasing transmural pressure. This process could trigger trigeminal nerve terminal that innervates the vascular wall, with consequent antidromal stimulation of sensory nerve endings at the level of hard vessels in the meninges, releasing the pro-inflammatory peptides (substance P and calcitonin gene-related peptide). Within this hypothetical scheme, serotonin released may also interact with trigeminovascular afferents to activate and/or facilitate the release of the neuropeptide at the level of the meningeal vessels trigeminal ganglion. The consequent dysregulation of the physiological negative feedback of serotonin on the orexinergic neurons would contribute to an altered modulation of the whole system, which can alter the sleep–wake cycle.

Serotonin function has been long implicated in the pathophysiology of migraine (60, 61). However, the underlying mechanisms of this correlation are not fully clarified yet. It has been hypothesized (62) that a massive release of serotonin from the MR nucleus would be implicated in the activation of serotonergic receptors located on the walls of large cerebral vessels. This event could lead to abnormal vasodilatation increasing transmural pressure. Subsequently, the terminal trigeminal nerve is activated with consequent antidromal stimulation of sensory nerve endings at the level of hard vessels in the meninges, generating the release of pro-inflammatory peptides (substance P and CGRP). Within this hypothetical scheme, serotonin released may also interact with trigeminovascular afferents to activate and/or facilitate the release of the neuropeptide at the level of the trigeminal ganglion. In light of the mutual regulation of these two systems, serotonergic and orexinergic, it is plausible that at the basis of this serotonergic pathway dysfunction, there may be an alteration of the orexinergic system. This alteration, therefore, would cause the cascade of events leading to the onset of migraine pain and to the dysregulation of the sleep/wake cycle (Figure 1).

## Discussion and Empirical Data

Migraine is a chronic neurovascular disease characterized by recurrent headache associated with autonomic, gastrointestinal, and focal neurological symptoms, and it is often associated with mood disorders and sleep disturbances. Migraine is a public health problem with great impact on both patients and society. Prevalence is estimated to be about 30% for women and 25% for men (63). In addition, this disease also affects pediatric age, with a prevalence of 1–3% between 3 and 7 years, 4–11% between 7 and 11 years, and 8–28% of adolescents (13–18 years) (64). Migraine is classified as one of the most disabling chronic disorders of the World Health Organization. The annual cost of loss-related productivity associated with migraine is heavy and has been estimated as the most expensive neurological disorder in Europe (65). Although migraine is a neurological disorder that has been studied for many years, many aspects of its neurophysiopathology remain to be clarified. Among the neurobiological systems that could be implicated in the migraine etiopathogenesis, both serotonergic and orexinergic systems have been considered in numerous studies. These same systems could be involved in the onset of sleep disorders. Biochemical, genetic, and pharmacological studies have investigated potential dysfunction of serotonergic system in the migraine. For example, as previously described, 5-HT plasma levels are reduced in patients with migraine compared to controls, during attack-free periods (66, 67); in addition, the urinary excretion of 5-HIAA (the most important serotonin metabolite) decreases with the frequency of migraine attacks (68). Moreover, in the scientific literature, it is reported that platelets of migraineurs showing qualitative differences in their serotonin released reaction and clumping (69). Even neuroimaging studies (particularly PET studies) suggest that cerebral synthesis of serotonin can be quantitatively altered in migraineurs (70). The most commonly used drugs for treating migraine attacks are the triptans, selective agonists for serotonin 5-HT1B and 5-HT1D receptors. It was hypothesized that triptans could reduce migraine pain decreasing serotonin brain synthesis (71). In general, the very dense innervation of thalamic trigeminovascular neurons can provide an anatomical substrate for a predominantly inhibitory effect of serotonin on transmission of trigeminovascular information between the thalamus and the cortex, as well as the inhibition of trigeminovascular thalamic neurons by local administration of 5HT1 agonists (52, 72). The total absence of 5HT1D receptors in the thalamus region may suggest that inhibition of thalamic trigeminovascular neurons response to dural stimulation occurs at an earlier synapse along the trigeminovascular pathway (73). Serotonergic neurons located in the MR nucleus are also involved in the regulation of the sleep/wakefulness cycle. As previously described, the destruction of these neurons or the administration of the inhibitor p-chloro-phenylalanine generates the insomniac habit that disappears restoring the synthesis of 5HT. The activity of serotonergic neurons in raphe dorsal nucleus is higher in wakefulness, lower during SWS, and almost quiescent during REM stages (74–77).

Specifically, orexins/hypocretins can regulate multiple homeostatic processes, including reward and arousal/wake state, exciting serotonergic DR and MR neurons (78, 79).

However, in which manner the serotonin regulates sleep/wake is still unclear. This neurotransmitter promotes waking and inhibits REM sleep in some cases, but it may also act as a sleep activator (80). These conflictual relationships could be due to the interaction between serotonergic neurons and other neurons involved in sleep/wake regulation, such as orexin/hypocretin neurons in the hypothalamus, which densely innervate serotonergic neurons in the MR nucleus, activating them directly and indirectly by binding OX1R and OX2R (81, 82).

In addition, orexin/hypocretin neurons receive dense serotonergic innervation and are inhibited by 5HT *via* the 5HT1A receptor, activating the GIRK channels (83, 84). It is not clear yet, though, how this circuit functions to regulate the sleep/wake cycle. It was described that orexinergic and serotonergic neurons located in raphe nuclei are more active in waking and less active during sleep SWS and REM. Moreover, as demonstrated in animal models, there is a critical role of the DRN–amygdale pathway in the orexinergic suppression of cataplectic episode (85). However, the level of activation of orexin/hypocretin cells is not directly related only to the arousal state, but they are less active during quiet wakefulness and discharge in active waking (86, 87). Regarding the role of orexin/hypocretin in the migraine, various studies suggest that mutation of the gene that encodes proteins involved in the orexin/hypocretin system may play a role in the etiopathogenesis of migraine. In particular, it was described that the HCRTR1 gene (orexin/hypocretin receptor 1 gene) may be related to migraine (88) and that the 1246G/A polymorphism of the hypocretin receptor 2 (HCRTR2) gene is significantly associated with headache cluster (89). As already mentioned, the orexin/hypocretin system seems to play many biological functions, and it appears to be involved in the regulation of dietary behaviors and energy expenditure (90–94). Furthermore, it may be related to the obesity control mechanisms (95). It is interesting to note that many studies suggest that obesity is comorbid with headache in general and migraine in particular. The obesity seems to be a risk factor for migraine progression and for migraine frequency both in adults and in children (96–101). Moreover, considering the complexity of the migraine symptom cascade (especially prodromal and postictal phases), the massive involvement of multiple cortical, subcortical, diencephalic, and brainstem structures is evident (102), making it much more than a simple headache (103).

Finally, we cannot omit that the orexin/hypocretin system is relevant in wakefulness and pain perception and integration (104), with an activation linked to the circadian periodicity (105). The orexin/hypocretin release levels are higher during the early day and lowest during the night (rest) (106), showing an evident role in sleep–wake regulation. When in humans and other mammalians, this complex system is dysregulated or disrupted, the narcoleptic syndrome can emerge with the relevant fragmented sleep–wake cycle and increase in the migraine prevalence (107).

Understanding the complex mechanisms underlying migraine and sleep disorders and how these mechanisms can interact with each other could pave the way for studies of new therapeutic strategies. Considering the social and economic implications of this disease on the world’s population, further studies are needed.

## Author Contributions

AnMe, IB, FP, MR, and LP conceived the study and participated in its design. MaSa, GiMe, AV, AnMe, MoSa, GaMa, CZ, and RM contributed to the conception and design. IV, AgMa, FS, GDA, and VM wrote the manuscript. DI, FL, SML, BG, RT, MM, GC, and MC drafted the article and revised it critically for important intellectual content; GiMe and MC made final approval of the version to be published. All the authors read and approved the final manuscript.

## Conflict of Interest Statement

The authors declare that the research was conducted in the absence of any commercial or financial relationships that could be construed as a potential conflict of interest.
